# MAPK cascades: key regulators of fruit quality traits

**DOI:** 10.1007/s00299-026-04004-3

**Published:** 2026-11-05

**Authors:** Ahmed Alabd

**Affiliations:** Department of Pomology, Faculty of Agriculture, Alexandria University, Alexandria, 21545 Egypt

**Keywords:** Fruit crops, Fruit disease resistance, Mitogen-activated protein kinase, MAPK cascades, Phosphorylation, Quality traits

## Abstract

Fruit quality is both a vital breeding goal and a consumer request. It is a sophisticated combination of several connected traits. These traits are highly regulated by various regulatory networks in which Mitogen-activated protein kinase (MAPK) cascades make essential contributions. MAPK cascades integrate diverse external and internal cues to regulate many biological processes related to fruit quality traits. They regulate fruit quality through the phosphorylation of downstream signaling targets, including structural proteins, enzymes, and transcription factors, thereby influencing the expression of quality traits-related genes. There is emerging compelling evidence to document that MAPK cascades play important roles in regulating fruit quality traits. However, a comprehensive review highlighting the major advances of recent years is notably lacking. This review explores the diverse regulatory proteins related to fruit quality traits that are targeted by MAPK proteins. In addition, this review highlights the current understanding of the key role of MAPK cascades in regulating several important quality traits and fruit disease resistance in major fruit crops. The involvement of MAPKKK–MAPKK–MAPK cascades in crucial processes related to fruit quality traits is also discussed. Finally, this review proposes future research directions to advance the broader understanding of MAPK-mediated regulation of fruit quality traits. Integrating single-cell and multi-omics approaches could elucidate MAPK regulatory networks and identify direct downstream substrates. Precision genome-editing tools, including base and prime editing, could also target validated MAPK phosphorylation sites for targeted improvement of fruit quality. Deeper insights into MAPK functions may facilitate novel strategies to improve fruit quality and enhance agricultural sustainability.

## Introduction

To face the growing challenges posed by increasing population growth and global climate change, fruit quality improvement and shelf-life extension of fruit crops should be significantly improved in the future (Tang and Chu 2017; Wang et al. 2019; ter Steeg et al. 2022; Hu et al. 2025). Achieving this goal relies heavily on biotechnological advancements and genetic crop improvement (Fernie et al. 2006; Bailey-Serres et al. 2019; De Mori and Cipriani 2023; Wang et al. 2023). A critical requirement for genetic crop improvement is the introduction of a new trait into crop lines of interest, which can be accomplished by introducing single or multiple genes via genetic engineering (Lombardo et al. 2016; Tang and Chu 2017; Gao 2021). In light of this, thousands of genes tightly linked to various quality traits have been identified and characterized (Alabd et al. 2024a, b, c). However, the functions of these genes are not regulated independently, but are coordinated through complex intracellular signaling networks that integrate developmental and environmental cues. Among these regulatory systems, mitogen-activated protein kinase (MAPK) cascade, a type of highly conserved signal transduction pathway, integrates diverse external and internal signals to control vital biological processes (Liu et al. 2007; Meng and Zhang 2013; Zhang and Zhang 2022; Khan et al. 2024). In this process, MAPKs recognize substrate proteins, including enzymes, structural proteins, and transcription factors (TFs), and phosphorylate them, thus playing a critical role in various signaling networks (Dóczi and Bögre 2018; Kumar et al. 2020; Roychowdhury et al. 2024; Shi et al. 2025). A typical MAPK signaling cascade consists of three components: a MAPKK kinase or MEK kinase (MAPKKK), MAPK kinase (MAPKK), and MAPK, which are sequentially activated through phosphorylation, ultimately enabling MAPKs to phosphorylate downstream substrate proteins (Zhang et al. 2015; Shi et al. 2025). MAPKs are activated by upstream MAPKKs, which are in turn phosphorylated and activated by MAPKKKs (Zhang et al. 2018; Zhang and Zhang 2022; Manna et al. 2023).

In recent decades, extensive studies have demonstrated that MAPK cascades play versatile roles in various aspects of the intracellular processes related to plant growth, development and stress responses (Tena et al. 2001; Zhang et al. 2006; Jiang et al. 2022; Wu and Wang 2024; Song et al. 2025). Researchers have also extensively characterized the biological functions of dozens of MAPK cascades, providing valuable information regarding the underlying biological processes related to fruit quality traits and fruit disease resistance (Wu et al. 2021, 2023; Zhang et al. 2021; Wei et al. 2023). Unlike previous reviews that have broadly discussed MAPK signaling in plant development, hormone signaling, and stress responses (Zhang et al. 2006; Pitzschke et al. 2009; He et al. 2020; Wu and Wang 2024), this review highlights recent advances in our understanding of MAPK-mediated regulation of fruit quality traits in major fruit crops, with particular emphasis on the molecular targets and mechanisms underlying specific quality traits. The review also provides an integrated and trait-specific perspective on the roles of MAPK proteins in regulating fruit pigmentation, sugar accumulation, softening, flavor metabolism, ripening, senescence, and postharvest disease resistance. In addition, key knowledge gaps are identified, and MAPK-specific future directions are proposed, including single-cell approaches, phosphoproteomics, multi-omics integration, and precision genome editing of validated phosphorylation sites. Finally, this work clearly demonstrates the potential for exploiting both MAPK cascades and their target proteins for fruit crop improvement.

## MAPKs fine-tune diverse regulatory proteins

MAPKs target substrate proteins to function as key regulators of quality traits in fruit crops. These target proteins comprise a diverse range of structural proteins and regulatory proteins. Several studies have provided evidence for direct MAPK-mediated phosphorylation of regulatory proteins. For example, MAPK3 and MAPK6 phosphorylate NAC (NAM, ATAF1/2 and CUC2) TFs, thereby modulating their activity in ethylene-mediated fruit softening (Li et al. 2023; Wei et al. 2023). Similarly, MAPK3, MPK14, and MPK4 have been implicated in the phosphorylation-dependent regulation of MYB TFs involved in fruit ripening and anthocyanin biosynthesis (Yang et al. 2021, 2022; Mao et al. 2022). Members of the basic leucine zipper (bZIP) family have also been identified as downstream substrates of MPK6-3, MPK6, and MAPK11-3, contributing to the regulation of fruit ripening and anthocyanin accumulation (Liang et al. 2020; Wu et al. 2021; Xing et al. 2023). Likewise, WRKY TFs have been reported to undergo MAPK3- or MAPK4-mediated phosphorylation, linking MAPK signaling to fruit ripening and anthocyanin accumulation (Chen et al. 2023; Zhang et al. 2024). In contrast, some reports indicated that MAPKs target regulatory proteins through protein–protein interactions or genetic associations. For example, MPK14 interacts with Brassinazole-resistant (BZR) TFs to modulate cell-wall-modifying genes during fruit ripening (Shan et al. 2020). Similarly, ethylene response factor (ERF) TFs have been identified as downstream components of MPK4- or MPK8-mediated pathways associated with fruit peel degreening and disease resistance (Wang et al. 2022c; Deng et al. 2024), while MAPK8-associated regulation of auxin response factors (ARFs) has been implicated in auxin- and ABA-mediated control of ascorbic acid accumulation (Xu et al. 2022). MPK6 has also been associated with MYC-type TFs in the regulation of methyl jasmonate-induced carotenoid biosynthesis and fruit coloration (Yue et al. 2022). MAPK-mediated regulation is not restricted to regulatory proteins, but can also involve direct modification of structural proteins. For example, in strawberry, FvMAPK3 repressed anthocyanin accumulation at low temperatures by phosphorylating and enhancing the degradation of FvCHS1 (Chalcone Synthase 1), an important structural protein that controls strawberry fruit ripening (Mao et al. 2022). These findings reveal that MAPKs play an important role in regulating fruit quality traits through phosphorylation and interactions with downstream targets. However, to date, only a few of the target proteins of MAPKs have been characterised in terms of their function. A comprehensive MAPK–target regulatory network remains largely unknown and requires further investigation.

## MAPKs coordinate fruit quality traits

**Fig. 1:**
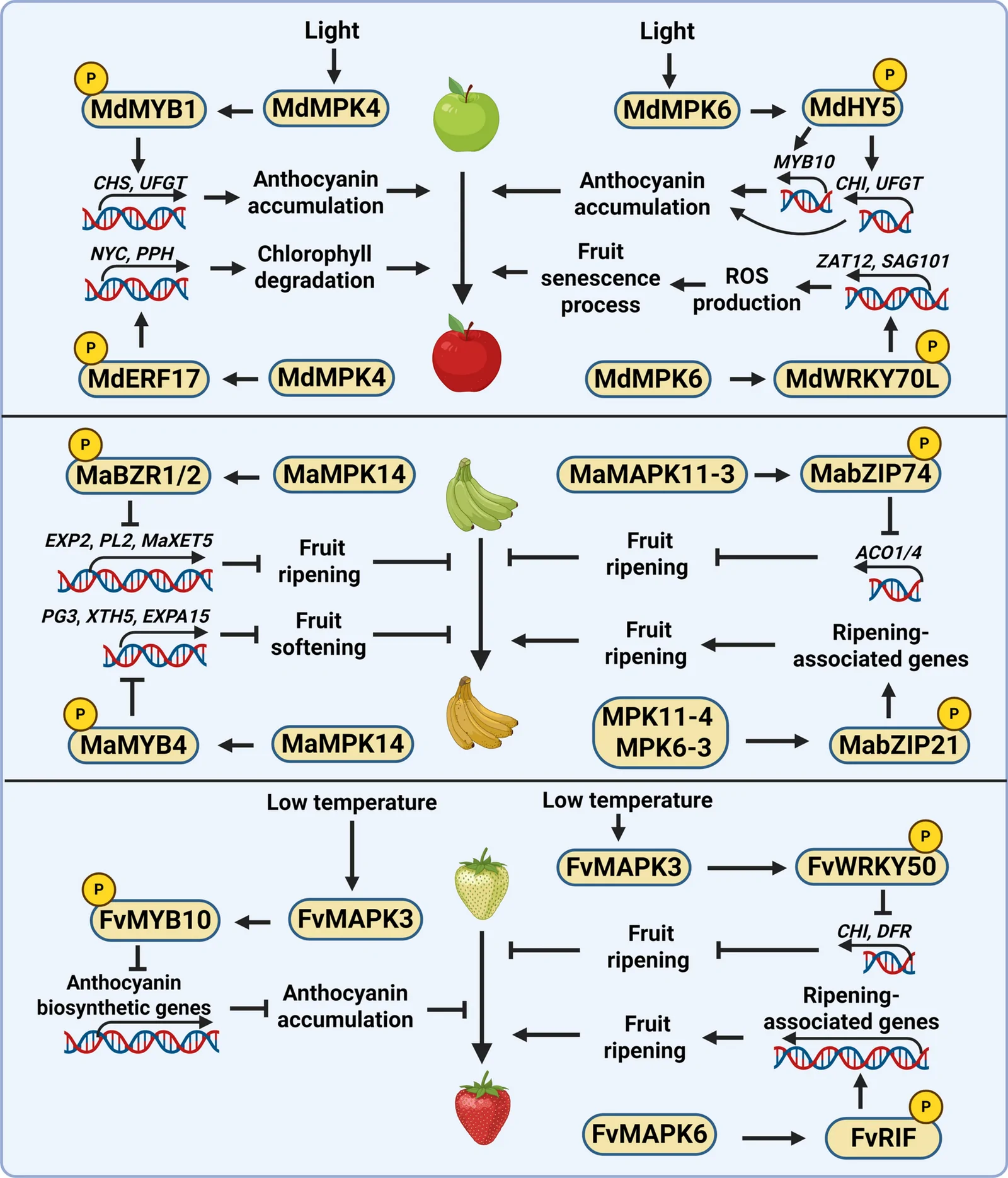
MAPKs coordinate different quality traits of fruit crops. In apple, some Fig. 1: MAPKs target anthocyanin regulatory proteins, such as MYB1 and HY5, which mediate anthocyanin accumulation in response to light. Other MAPKs target regulatory proteins, such as ERF17 and WRKY70L, which are involved in chlorophyll degradation and fruit senescence process, respectively. In banana, some MAPKs phosphorylate several regulatory proteins to regulate fruit ripening. In strawberry, MAPK3 targets MYB10 to regulate anthocyanin accumulation in response to low temperature, while MAPK3 and MAPK6 are involved in regulating fruit ripening

In fruit crops, MAPKs play important roles in growth and development, defense signaling, hormone signaling (Smékalová et al. 2014; He et al. 2020; Xie et al. 2023). Research in recent years also established indispensable roles for MAPKs in coordinating several physiological processes related to diverse quality traits (Fig. 1) (Zhang et al. 2013; Asif et al. 2014; Shu et al. 2023). However, their pleiotropic functions may impose potential trade-offs, whereby manipulation of a MAPK to enhance a specific quality trait could concomitantly affect other desired traits. For example, in strawberries, FvMAPK6 improves fruit quality, including both sugar and anthocyanin accumulation, but it significantly reduces fruit yield and vegetative growth (Feng et al. 2026). Therefore, exploiting MAPKs for fruit quality improvement requires careful consideration of their tissue- and developmental stage-specific functions, upstream regulatory inputs, downstream substrates, and potential unintended phenotypic effects. This section focuses on the functions of MAPK proteins in regulating the quality traits of various fruit crops.

### MAPKs regulate pigment and sugar accumulation

Pigment accumulation enhances the nutritional and commercial value of fruit crops (Alabd et al. 2024c). As important sources of natural antioxidants, fruit pigments contribute to human health, while serving as key determinants of fruit coloration, quality, and consumer acceptance (Alabd et al. 2024c). Evidence across several species reveals that MAPK proteins fine-tune pigment accumulation by phosphorylating key TFs. In strawberry, FvMAPK6 phosphorylates FvRIF, a NAC TF, which directly regulates key genes in the anthocyanin biosynthetic pathway, thereby promoting anthocyanin accumulation (Li et al. 2023). MAPK proteins also integrate environmental cues, such as temperature and light to fine-tune pigment biosynthesis. For example, low temperature induces FvMAPK3 to repress anthocyanin accumulation in strawberri by targeting several proteins. FvMAPK3 suppresses anthocyanin accumulation in response to low temperature by phosphorylating FvMYB10 and FvCHS1, thereby reducing FvMYB10 transcriptional activity and promoting FvCHS1 proteasomal degradation, respectively (Mao et al. 2022). Under low temperature conditions, FvMAPK3 also phosphorylates and degrades FvWRKY50 to reduce its transcriptional activity on anthocyanin-related genes, thereby suppressing anthocyanin accumulation (Chen et al. 2023). Moreover, under high-light conditions, SmMPK4.1 negatively regulates anthocyanin accumulation by inducing the transcriptional activation of SmMYB75 on *SmCHS* expression in eggplant (Liao et al. 2024). In contrast, in apple, light signaling activates two major axes, MdMAPK4-MdMYB1 and MdMAPK6–MdHY5 to promote light-induced anthocyanin accumulation. Under light conditions, activated MdMPK4 targets MdMYB1 through a direct interaction, inducing its phosphorylation. This posttranslational modification stabilizes the MdMYB1 protein, resulting in the activation of downstream genes that contribute to anthocyanin biosynthesis (Yang et al. 2021). Moreover, light-activated MdMPK6 phosphorylates MdHY5, leading to the accumulation of phospho-MdHY5, which enhances MdHY5’s binding affinity for its target anthocyanin-associated genes (Xing et al. 2023). Additionally, dark-induced MdMAPK4 phosphorylates MdERF17, which regulates chlorophyll degradation-related genes during light/dark transitions (Wang et al. 2022c), indicating that the same MAPK protein can integrate distinct transcriptional programs associated with different aspects of fruit coloration. Therefore, these findings suggest that substrate selection by a single MAPK may provide a mechanism for coordinating multiple developmental and metabolic processes within the same fruit crop.

In addition, MAPK proteins can function as important phosphorylation hubs that coordinate both sugar and anthocyanin accumulation in response to sucrose signaling. In strawberry, FvMAPK6, induced by sucrose treatment, directly phosphorylates the transcription factors FvMYB44.1 and FvMYB44.2, which function as repressors of sugar and anthocyanin accumulation. This phosphorylation reduces the stability and transcriptional activity of these proteins, attenuates their repression of downstream target genes, such as *FvCHI*, *FvSPS3* and *FvSWEET1*, thereby coordinating anthocyanin and sugar accumulation (Feng et al. 2026). Moreover, transient overexpression of *FaMAPK5* and *FaMAPK10* increases anthocyanin, total sugar, and glucose contents in strawberry fruit (Zhang et al. 2021). Collectively, these findings indicate that MAPK proteins act as key signaling nodes that integrate light, temperature, and sucrose signals to regulate pigment and sugar accumulation through phosphorylation of diverse regulatory targets. However, the mechanisms governing MAPK substrate specificity and signaling responses in the regulation of anthocyanin and sugar accumulation under different environmental conditions remain poorly understood.

### MAPKs regulate fruit softening and flavor metabolism

Fruit softening, a common physiological process in most fleshy fruits after harvest, is a major factor limiting postharvest life, handling, transportation, storage, and overall fruit quality. Growing evidence indicates that MAPK proteins are fully integrated in various regulatory networks to play key roles in regulating fruit softening. For example, in banana, MaMPK2 interacts with and phosphorylates MabZIP93 to promote MabZIP93-mediated transcriptional activation of cell wall modifying genes, such as *MaPL2*, *MaPE1*, *MaXTH23* and *MaXGT1* (Wu et al. 2019). In strawberry, FvMAPK6 phosphorylates FvRIF, which directly modulates core genes involved in fruit softening (Li et al. 2023). Moreover, FvMAPK3 induces the expression of softening-related genes, such as *FvXYL1*, *FvEXP1*, and *FvEXP2* (Mao et al. 2022); however, whether FvMAPK3 directly or indirectly regulates these genes needs to be explored further. In apple, ethylene-induced MdMAPK3 phosphorylates MdNAC72, resulting in suppression of its transcriptional repression activity on *MdPG1*, thereby contributing to ethylene-induced fruit softening (Wei et al. 2023). In fig (*Ficus carica* L.), FcMAPK4-phosphorylated FcNOR activates FcERF5 to promote fruit softening through activation of *FcPG12* expression (Wang et al. 2026). In contrast, in banana, MaMPK14 interacts with and phosphorylates MaMYB4 and MaBZR1/2, enhancing their transcriptional repression of genes associated with fruit softening (Shan et al. 2020; Yang et al. 2022). MaMPK14 induces phosphorylation of MaBZR1/2 through a direct interaction, and this phosphorylation strengthens the MaBZR1/2-mediated transcriptional inhibition of cell-wall-modifying genes, such as *MaEXP2*, *MaPL2* and *MaXET5* (Shan et al. 2020). Moreover, through direct interaction, MaMPK14 also phosphorylates MaMYB4 to enhance the MaMYB4-mediated transcriptional inhibition of genes associated with ethylene biosynthesis and fruit softening, such as *MaACS1*, *MaXTH5*, *MaPG3*, and *MaEXPA15* (Yang et al. 2022). Thus, MaMPK14 functions as a signaling integrator linking MAPK activity with ethylene- and brassinosteroid-responsive transcriptional networks during postharvest ripening. Recently, it was found that MAPK proteins can function in the regulation of flavor metabolism. FvMAPK6 phosphorylates FvJAZ12 to alleviate its repression of FvMYC2, promoting the expression of *lipoxygenase 3 (FvLOX3)*, an important gene implicated in regulating JA biosynthesis and flavor-associated metabolism, thereby linking MAPK activity to jasmonate biosynthesis and flavor-associated metabolism (Wang et al. 2024a). Collectively, these findings indicate that MAPK proteins play important roles in fruit softening by regulating cell-wall-modifying genes through phosphorylation of diverse transcriptional regulators and by integrating hormonal signals, particularly ethylene and brassinosteroids. In contrast, evidence linking MAPK signaling to flavor metabolism remains scarce, with only limited studies demonstrating its involvement in jasmonate signaling and flavor-related metabolic processes. Further identification, validation and comprehensive analysis of additional MAPK proteins, will provide effective strategies for modulating flavor quality and softening of fruit crops.

### MAPKs regulate fruit ripening and senescence

Fruit ripening is a complex and genetically programmed process orchestrated by a network of TFs (Zhang et al. 2025; Saeed et al. 2026), whose functions are regulated by MAPK proteins through phosphorylation. For example, MaMAPK11-3 interacts with and phosphorylates MabZIP74, thereby attenuating MabZIP74-mediated transcriptional repression of the ethylene biosynthetic genes *MaACO1* and *MaACO4* during banana fruit ripening (Liang et al. 2020). Furthermore, MaMPK6-3 directly interacts with and phosphorylates MabZIP21, and that phosphorylation of MabZIP21 increases its transcriptional activation activity on the ripening-related genes (Wu et al. 2021). Moreover, MaMAPK1 enhances MaNAC29-mediated transcriptional activation of *MbACO2*, thereby promoting ethylene production during banana fruit ripening (Tang et al. 2023). In strawberry, FaMAPK19 has also been identified as a regulator of fruit ripening, as manipulation of FaMAPK19 expression affects the expression of genes implicated in fruit ripening, such as *FaCS1*, *FaCS2*, *FaEXP1*, *FaEXP2*, *FaEXP4*, and *FaEXP5* (Zhang et al. 2020). Moreover, FaMAPK5 and FaMAPK10 are involved in regulating fruit ripening by increasing the content of anthocyanin, sugar and ABA (Zhang et al. 2021). In addition, FvMAPK6 interacts with and phosphorylates FvRIF, which directly regulates the expression of genes encoding TFs involved in fruit ripening (Li et al. 2023). In tomato, SlMAPK4 inhibits fruit ripening by phosphorylating SlWRKY6, which downregulates the expression of *SlSGR1* and *SlSAG12* (Zhang et al. 2024). In apple, MdMAPK6 phosphorylates MdWRKY9 at the Tyr394 site, enhancing the stability and activity of MdWRKY9, which binds to the inhibitor MdERF5L, thereby attenuating MdERF5L-mediated regulation of downstream *MdACS1* and promoting fruit ripening (Wang et al. 2025b). In papaya (*Carica papaya*), CpMAPK20 displayed significant induction in fruit flesh by ethephon treatment, suggesting its potential involvement in fruit postharvest ripening through an ethylene signaling pathway or the regulation of cell wall metabolism (Zhu et al. 2024). In addition to ripening, fruit undergoes senescence, which is an inevitable aspect of the fruit life cycle. This process directly affects fruit quality, pathogen resistance, and shelf life, thereby contributing to the loss of the economic value of postharvest fruit. It has recently been demonstrated that MdMPK6/02G phosphorylates MdWRKY70L, enhancing its stability. This modification promotes the expression of the downstream senescence-related genes *MdSAG101* and *MdZAT12*, leading to increased ROS accumulation and ultimately causing fruit senescence and browning (Wang et al. 2025a). Collectively, these findings indicate that MAPK proteins play important roles in regulating fruit ripening and senescence by phosphorylating diverse TFs and modulating ethylene biosynthesis, ripening-related gene expression, and ROS accumulation. However, the mechanisms underlying MAPK substrate specificity and their differential regulation of ripening and senescence remain poorly understood. Future studies should identify additional MAPK substrates and phosphorylation sites and clarify how MAPK signaling integrates hormonal and ROS-related pathways during fruit ripening and senescence. Such knowledge could provide new opportunities for targeted modulation of ripening and senescence to modulate fruit quality and postharvest life.

## Role of MAPKs in postharvest fruit disease resistance

**Fig. 2:**
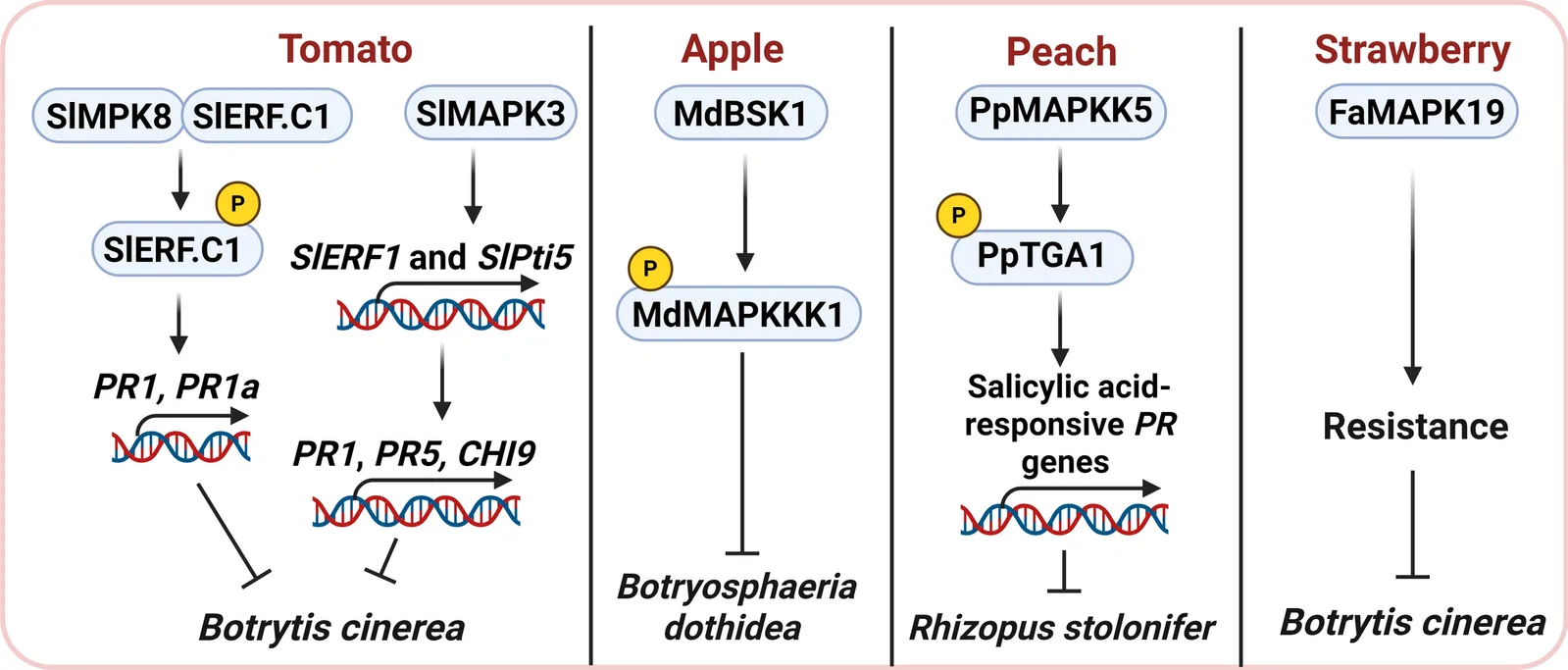
Biological functions of MAPKs in fruit disease resistance among crops. In tomato, MPK8 and MAPK3 enhance resistance against *Botrytis cinerea* by indirectly modulating the expression of *PR* genes. In apple, MdBSK1 modulates defense against *Botryosphaeria dothidea* by targeting MdMAPKKK1. In peach, PpMAPKK5 targets PpTGA1, upregulating the salicylic acid-responsive *PR* genes, thereby enhancing defense against *Rhizopus stolonifer*. In strawberry, FaMAPK19 positively affects resistance against *Botrytis cinerea*

Fruits are vulnerable to pathogen assaults during postharvest stages that affect their quality and storability, resulting in huge economic losses (El Oirdi et al. 2011; Alkan and Fortes 2015). In response to a pathogen attack, fruit utilize diverse resistance pathways to resist against pathogen invasion at multiple levels in which MAPKs make essential contributions (Fig. 2) (Mansoor et al. 2024; Li et al. 2025). For example, SlMPK8 interacts with and phosphorylates SlERF.C1. The phosphorylation of SlERF.C1 enhances its interaction with *PR1* and *PR1a* genes, thereby increasing the transcription of these two key *PR* genes. Consequently, tomato fruit becomes more resistant to *Botrytis cinerea* (Deng et al. 2024). Moreover, recent study shows that yeast cell wall and β-glucan induce *SlMAPK3* expression to enhance tomato fruit against *B. cinerea*. Induced *SlMAPK3* directly regulates the transcription of *SlPti5* and *SlERF1*, which in turn upregulates the transcription of *PR* genes (*SlCHI9*, *SlPR1* and *SlPR5*), resulting in enhanced resistance against *B. cinerea* (Guo et al. 2021). Incidentally, inhibition of *SlMPK1/2/3* disrupts tomato fruit defense signaling pathways and enhances the susceptibility to *B. cinerea* (Zheng et al. 2015). In walnut, JrMAPK3 interacts with and phosphorylates JrWRKY22, enhancing its transcriptional activity on the promoters of the pathogenesis-related genes *JrPR1* and *JrGLU*. This promotes their expression and enhances resistance to *Colletotrichum gloeosporioides*, thereby increasing anthracnose resistance (Xu et al. 2026). In apple, acibenzolar-S-methyl (ASM) induce fruit resistance to *Penicillium expansum* by promoting the expression of *MdMAPK2*, *MdMAPK4*, and *MdMAPKK1*, and suppressing the expression of *MdMAPK3* (Cheng et al. 2020). MdMAPKKK1 positively regulates apple resistance to *Botryosphaeria dothidea* and this regulation depends on the phosphorylation of MdMAPKKK1 by MdBSK1 (Wang et al. 2022b). GABA (γ-Aminobutyric acid) enhances disease resistance against *Gilbertella persicaria* by regulating the expression of *HcMAPK1/7*, *HcMAPKK2/4* and *HcMAPKKK1* in red pitaya fruit (Ding et al. 2023). In peach, PpMAPKK5 positively involved in induced resistance against the fungus *Rhizopus stolonifer* through its interaction with PpTGA1, which in turn upregulates the expression of salicylic acid (SA)-responsive *PR* genes (Li et al. 2021). PpMAPK1 is involved in β-aminobutyric acid (BABA)-induced resistance to *R. stolonifer* in postharvest peach fruit. Functional analyses demonstrated that the PpMAPK1 positively regulate SA-dependent defense gene expression (*PpNPR1*, *PpPR1*, *PpBGL2*, and *PpPR5*) by coordinating with SNARE13 in a Nonexpressor of pathogenesis-related genes 1 (NPR1)-dependent manner, thereby contributing to the enhancement of defense responses against *R. stolonifer* in postharvest peach fruit (Li et al. 2024). In strawberry, overexpression of *FaMAPK19* improved disease resistance to *B. cinerea*, but antisense expression of *FaMAPK19* caused a reduction in disease resistance. These results demonstrated that FaMAPK19 can enhance the resistance by influencing the expression of genes related to disease resistance and hormone metabolism (Zhang et al. 2020). In addition, FaMAPK5 positively improved the disease resistance of strawberry fruit against *B. cinerea* (Zhang et al. 2021). These findings highlight the critical roles for MAPKs in regulating fruit disease resistance across various species through diverse regulatory mechanisms, including regulation of hormone signaling and reprogramming of gene expression (for example, *PR* genes).

### MAPKKK–MAPKK–MAPK cascades in fruit crops

The MAPK cascade normally consists of three kinases, namely MPKKK, MPKK, and MAPK (Zhang et al. 2015; Shi et al. 2025). Compared to MAPK proteins, little is known about the role of MPKKK and MPKK in regulating fruit quality traits. For example, MdMKK9 induces anthocyanin accumulation in apple in response to low nitrogen stress by increasing the expression level of anthocyanin synthesis-related genes (Sun et al. 2022). In tomato, RNA-seq analysis indicated that SlMKK4 is responsible for pollen development by fine-tuning gene networks involved in auxin and sugar metabolism (Chen et al. 2024). In addition, ethylene-mediated regulation of organic acid metabolism in strawberry fruits through the FaMAP3K-FaERF13-FaKAT module. Ethylene suppresses the interaction between FaMAP3K (MAPKKK) and FaERF13 proteins, potentially relieving the transcriptional repression of FaKAT and contributing to decreased total organic acid accumulation during strawberry fruit ripening (Yu et al. 2026). Collectively, these findings indicate that MAPKKKs and MAPKKs can contribute to the regulation of fruit quality traits by modulating downstream transcriptional and metabolic processes, although their functions remain considerably less characterized than those of MAPKs.

**Fig. 3:**
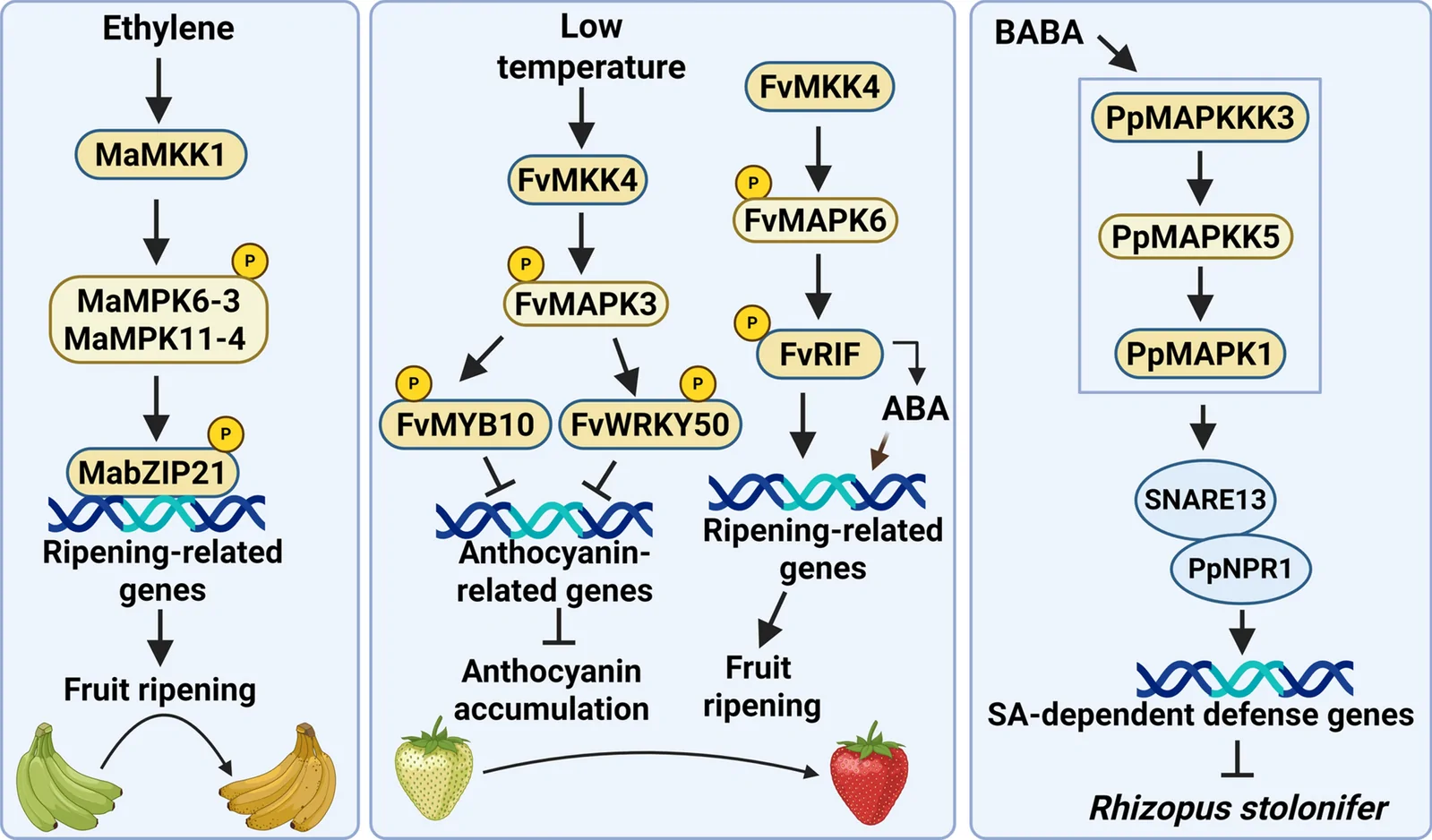
MAPKKK–MAPKK–MAPK modules implicated in regulating fruit quality traits. In banana, MaMKK1 induction by ethylene phosphorylates MaMPK6-3 and MaMPK11-4 to enhance bZIP21 phosphorylation, thereby activating ripening-related genes that contribute to banana fruit ripening. In strawberry, MKK4–MAPK3 model inhibits anthocyanin accumulation in response to low temperature by phosphorylating various regulatory proteins, such as MYB10 and WRKY50, while MKK4–MAPK6 model promotes strawberry fruit ripening by phosphorylating RIF. In peach, PpMAPKKK3–PpMAPKK5–PpMAPK1 module is involved in BABA-induced pattern-triggered immunity defense against *Rhizopus stolonifer* in postharvest peach fruit

Evidence across several species reveals that MAPKKK-MAPKK-MAPK cascades are responsible for various biological events. However, the regulatory effects of the MAPK cascade in fruit crops have just begun to be explored (Fig. 3). For example, in banana, the MaMKK1-MaMPK6-3/11–4 module is involved in banana fruit ripening. Specifically, MaMKK1, induced by ethylene, targets MaMPK6-3 and MaMPK11-4, phosphorylating them at the pTEpY residues, resulting in MPK activation. The activated MaMPK6-3 and MaMPK11-4 phosphorylateMabZIP21, an important ripening regulator, to enhance MabZIP21 transcription activity of ripening-associated genes that contribute to banana fruit ripening (Wu et al. 2023). In strawberry, FvMKK4-FvMAPK3 module is responsible for the low-temperature-mediated repression of anthocyanin accumulation, partially through phosphorylating FvWRKY50 and inducing its protein degradation by ubiquitination (Chen et al. 2023). In another study, it was reported that the FvMKK4-FvMAPK3 module represses FvMYB10-mediated anthocyanin biosynthesis by decreasing its transcriptional activity at low temperatures (Mao et al. 2022). Recently, it was reported that the MKK4-MAPK6 module is involved in strawberry fruit ripening. FvMKK4 interacts with and phosphorylates FvMAPK6 to promote the transcriptional activity of FvRIF. Activated FvRIF enhances anthocyanin accumulation directly by regulating the structural genes or indirectly by activating *MYB10* expression during strawberry fruit ripening (Li et al. 2023). Considering that MKK4-MAPK6-mediated phosphorylation activates FvRIF, which in turn positively regulates FvMYB10, it is reasonable to speculate that MKK4-MAPK6 module plays a positive role in controlling *FvMYB10* expression. However, whether MKK4-MAPK6 module directly mediates phosphorylation of FvMYB10 and how it affects FvMYB10 activity remain unclear. Since FvMKK4-FvMAPK3-mediated phosphorylation negatively regulates FvMYB10 (Mao et al. 2022), it seems likely that MKK4-MAPK6 module antagonizes FvMKK4-FvMAPK3 module in the regulation of FvMYB10, illustrating the complexity and rigorousness of MAPK cascades-mediated regulation of their substrate proteins. However, further investigation of the interplay between these two modules is required.

In postharvest peach fruit, the PpMAPKKK3–PpMAPKK5–PpMAPK1 module participates in BABA-induced pattern-triggered immunity defense against *R. stolonifer*. PpMAPK1 acts as downstream partners of PpMAPKK5 and PpMAPKKK3 to positively regulate SA-dependent defense gene expression by coordinating with SNARE13 in an NPR1-dependent manner, thereby contributing to the enhancement of defense responses in postharvest peach fruit (Li et al. 2024). These studies highlight the involvement of MAPK cascades in crucial processes related to fruit quality traits. However, discovering new MAPK cascades, along with advanced genome-editing tools, will place MAPK cascades at the forefront of future fruit crop resistance breeding strategies.

## Conclusions and future perspectives

In fruit crops, several studies have highlighted the promising role of MAPK proteins in enhancing fruit quality formation. As a result, research on the mechanistic insights into the functions of MAPK proteins has recently escalated. Nevertheless, several structural proteins, TFs, and other components in diverse fruit crop species still remain to be explored as possible downstream substrates of MAPK proteins. Additionally, how MAPK proteins integrate diverse biological signaling events related to fruit quality formation, such as hormone signaling, light signaling, and defense signaling, remains to be fully elucidated. With the advent of next-generation and third-generation sequencing techniques, it is now possible to sequence the entire genome of various crop species, paving the way for comparative analyses of MAPK protein sequences and activities (Manna et al. 2023). This will facilitate the identification of more MAPK candidates for fruit crop improvement programs. Furthermore, CRISPR-Cas9 mediated genome modification serves as an efficient technique for targeted mutagenesis to generate more useful engineered proteins (Xu et al. 2020; Zhou et al. 2020; Ma et al. 2023). However, future studies should move beyond broadly applicable genomic approaches and focus on MAPK-specific regulatory mechanisms. In particular, single-cell resolution approaches could be employed to map the spatial and temporal dynamics of MAPK signaling across distinct fruit tissues and developmental stages. Systematic application of phosphoproteomic approaches under diverse developmental and environmental conditions would also facilitate the identification and validation of direct MAPK downstream substrates and phosphorylation targets. Integrating phosphoproteomics with transcriptomics, metabolomics, proteomics, and other multi-omics approaches could further enable the construction of comprehensive MAPK-centered regulatory landscapes underlying fruit quality formation. Moreover, genome-editing strategies should extend beyond simple gene knockouts to include base editing and prime editing for the precise introduction of phospho-mimetic or phospho-dead mutations at validated MAPK phosphorylation sites. Such targeted modifications could provide greater mechanistic insight into MAPK-mediated regulation and enable more predictable and precise phenotypic enhancement of fruit quality traits. Therefore, the application of these MAPK-specific approaches is expected to significantly expand the functional understanding and breeding applications of MAPKs in fruit crops.

Until now, the biological significance of several MAPK-target proteins from various fruit crops has been revealed. It is evident that MAPKs play a variety of roles in the intricate regulatory networks associated with fruit quality formation. A deeper understanding of these functions within these networks will probably allow breeders to choose appropriate targets for modulating fruit quality traits. Functional validation of more target proteins of MAPKs in such networks will be one of the major future challenges. As functional studies focus on conserved MAPKs, information regarding the essential roles of many crop-specific MAPKs is extremely limited. Furthermore, the involvement of MAPKs in various fruit quality traits, such as size, flavor, bioactive compounds, and texture, remains largely unknown in fruit crops. Notably, most known MAPKs related to quality traits come from apple, banana, and strawberry and very few MAPKs from other crops have been investigated. For these crops, much more work is needed to decipher the preliminary MAPK-mediated regulatory network.

In addition, a particularly critical direction for future studies is to elucidate the upstream MAPKKK–MAPKK signaling of MAPK proteins underlying fruit quality regulation. Although individual MAPK proteins and their downstream targets have been increasingly characterized, the corresponding MAPKKKs and MAPKKs that activate specific MAPK proteins in fruit crops remain poorly understood. Systematic identification and functional characterization of MAPKKK–MAPKK–MAPK cascades, including their phosphorylation relationships, tissue-specific expression patterns, and spatiotemporal activation dynamics, will be essential for establishing complete signaling cascades associated with fruit quality formation.

## Data Availability

No datasets were generated or analysed during the current study.
